# A First Report of Synchronous Intracapsular and Extracapsular Hepatic Adenoma

**DOI:** 10.1155/2017/9053568

**Published:** 2017-12-19

**Authors:** Murat Derebey, Ilhan Karabicak, Savas Yuruker, Gokhan Lap, Bilge Can Meydan, Murat Danaci, Kagan Karabulut, Necati Ozen

**Affiliations:** ^1^Department of General Surgery, Tatvan State Hospital, Bitlis, Turkey; ^2^Department of General Surgery, VM Medical Park Hospital, Samsun, Turkey; ^3^Medical Faculty, Department of General Surgery, Ondokuz Mayis University, Samsun, Turkey; ^4^Department of General Surgery, Adilcevaz State Hospital, Bitlis, Turkey; ^5^Medical Faculty, Department of Pathology, Ondokuz Mayis University, Samsun, Turkey; ^6^Medical Faculty, Department of Radiology, Ondokuz Mayis University, Samsun, Turkey

## Abstract

Although the gallbladder is the most common site of ectopic liver, it has been reported in many other organs, such as kidney, adrenal glands, pancreas, omentum, stomach, esophagus, mediastinum, lungs, and heart. Hepatocytes in an ectopic liver behave like normal hepatocytes; furthermore, they can be associated with the same pathological findings as those in the main liver. Ectopic liver in the gallbladder can undergo fatty change, hemosiderosis, cholestasis, cirrhosis, hemangioma, focal nodular hyperplasia, adenoma, and even carcinogenesis. The incidence of extracapsular hepatic adenoma is not known, but only two cases have been reported. Here, we provide the first case report of synchronous multiple intracapsular and extracapsular hepatic adenomas. A 60-year-old woman with multiple hepatic adenomas and one 7 × 5 × 5 cm ectopic hepatic adenoma attached to the gallbladder fundus complicated with abdominal pain is presented.

## 1. Introduction

Ectopic liver is a very rare entity in which the liver tissue is formed outside the liver with no hepatic connection [1]. Although the gallbladder is the most common site, it has been reported in many different organs such as kidney, adrenal glands, pancreas, omentum, stomach, esophagus, mediastinum, lungs, and heart [1–7].

Hepatocellular adenoma is a rare, benign liver neoplasm which is usually found in young women. Oral contraceptives, anabolic steroid usage, and glycogen storage disease are the well-known risk factors [8]. Although most series report solitary hepatic adenoma, two, three, or more adenomas have also been reported [8, 9]. The incidence of hepatic adenoma increased with the wide usage of the imaging modalities for nonspecific abdominal symptoms [10, 11].

The incidence of extracapsular hepatic adenoma is not known, but only two cases have been reported. Leone et al. [12] reported the first ectopic hepatic adenoma with a thin stalk attached to the liver, and Vargas-Flores et al. [13] reported the second ectopic hepatic adenoma attached to the gallbladder.

We report the first case of synchronous multiple intracapsular and extracapsular hepatic adenomas. A 60-year-old woman with three hepatic adenomas and one 7 × 5.5 cm ectopic hepatic adenoma attached to the gallbladder fundus that complicated with abdominal pain is presented.

## 2. Case Report

**Figure 1:**
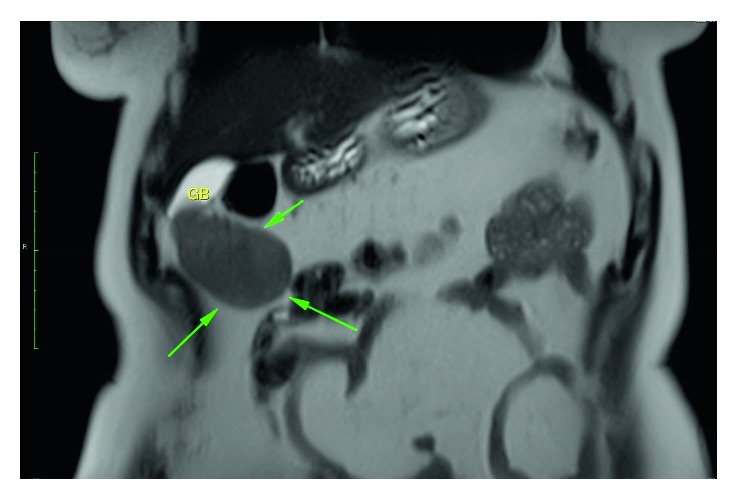
Coronal T2-weighted image. The mass extending from the gallbladder (arrows) and the gallbladder (GB) are seen.

**Figure 2:**
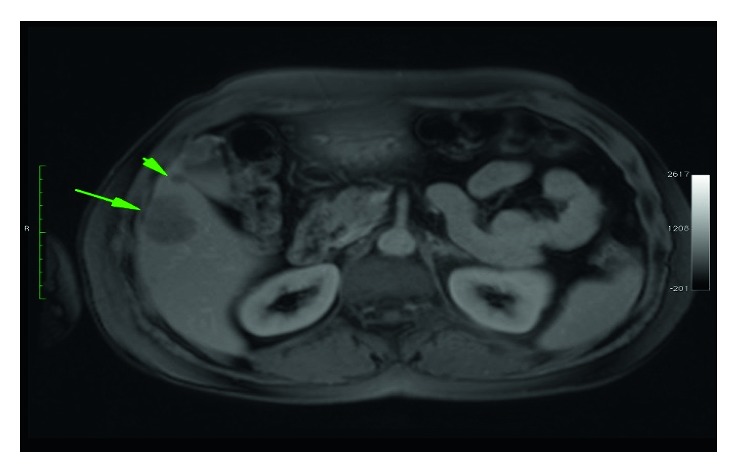
T1-weighted gradient-echo contrast-enhanced image. Two lesions are seen in the right liver lobe adjacent to the gallbladder.

A 60-year-old woman admitted to the general surgery department with nonspecific abdominal pain. On admission, she appeared obese with BMI 30.4. Physical examination was normal. Laboratory studies were within normal limits, including serum a-fetoprotein (AFP), CEA, CA 19-9, and HbA1c levels. An ultrasonography showed a 7 × 5 × 5 cm mass which attached to the gallbladder fundus and three small hypoechoic masses in segment 5 and two hypoechoic masses in segment 7. Magnetic resonance images showed lesions with markedly decreased signal intensities on T1-weighted and T2-weighted fat-saturated images. These findings are likely to contain microscopic fat. On dynamic contrast-enhanced MRI series, lesions markedly enhanced on arterial phase and showed wash out on delayed phase (Figures 1 and 2).

**Figure 3:**
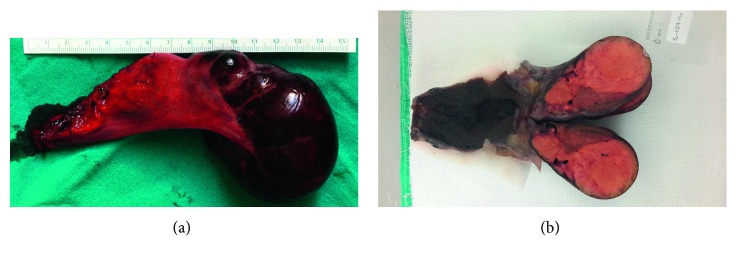
(a) and (b) Macroscopically, 7 × 5.5 × 4 cm ectopic liver adenoma in the gallbladder fundus serosa with no connection to the mother liver.

She was questioned for the risk factors for hepatic adenoma. No risk factors were identified other than obesity. An open cholecystectomy, liver segment 5-6 resection, and partial segment 7 resection were performed. Patology showed macroscopically, ectopic liver adenoma with no connection to the mother liver (Figures 3(a) and 3(b)) and eight hepatocellular adenomas, some of them are microadenomas (<5 mm), within the resected liver parenchyma. During microscopic examination of ectopic adenoma, the possibility of well-differentiated hepatocellular carcinoma (over 50 years of age, 7 cm adenoma size, and arising in the ectopic liver) was considered, but we did not find any suspicious morphology for carcinoma. Also, nuclear beta-catenin was negative.

**Figure 4:**
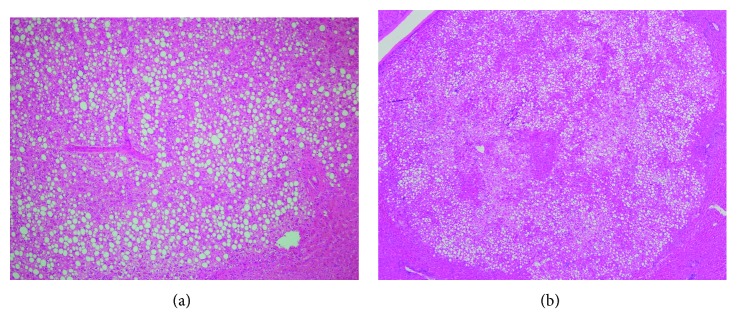
(a) Histologic findings of ectopic hepatocellular adenoma attached to the gallbladder serosa (H&E, ×100). (b) Steatotic microadenoma in the mother liver (H&E, ×40).

Histologic examination showed hepatic adenoma at the gallbladder and other three adenomas within the resected liver segments (Figures 4(a) and 4(b)). The postoperative course was favorable and uneventful. The patient was discharged on postoperative day 5. Additionally, she remains clinically and radiologically asymptomatic over 26 months after surgery.

## 3. Discussion

Ectopic liver tissue is an incidental finding during a laparoscopy, laparotomy, or autopsy [1]. Although the diagnosis rate of ectopic liver tissue attached to the gallbladder is increased in the laparoscopic era, it is reported between % 0.05 to 0.28 [14, 15]. The symptoms depend on the location and size of the ectopic liver tissue. Most of them are asymptomatic but can cause nonspesific abdominal pain with the growth of the ectopic liver tissue [16, 17].

Hepatocytes in an ectopic liver behave like normal hepatocytes and can show the same pathological findings as those of the main liver [1, 18]. Ectopic liver in the gallbladder can undergo fatty change, haemosiderosis, cholestasis, cirrhosis, haemengioma, focal nodular hyperplasia, adenoma, and even carcinogenesis [1, 12–15, 19–22].

Prognosis of hepatic adenoma is not well established. Therefore, management is aimed according to symptoms, size, number, location, and certainty of diagnosis [13]. The incidence and treatment of extracapsular hepatic adenoma is not known. So far, two cases of extracapsular liver adenoma have been reported which were big in size, were symptomatic, and required surgery [12, 13].

Treatment of the ectopic liver tissue in the gallbladder is controversial. It will be removed during cholecystectomy performed for symptomatic gallstones [1]. Leone et al. [12] reported that 27 of the 100 ectopic livers have developed hepatocellular carcinoma. Among the 27 cases of ectopic HCCs, liver cirrhosis was found in only 6 patients (25%). They conclude that the ectopic liver tissue is more prone to hepatocarcinogenesis than the mother liver [12]. Segura-Sánchez et al. [22] strongly suggests excision of the ectopic liver tissue diagnosed even during laparoscopies performed for other reasons.

In conclusion, ectopic hepatic adenoma is extremely rare. We report the first case of synchronous intracapsular and extracapsular hepatic adenoma. The natural course of ectopic liver tissue is unpredictable. Ectopic liver tissue can show the same pathological findings as those of the main liver. The carcinogenesis and adenoma transformation should be kept in mind during the follow-up if seen incidentally during other procedures.
